# A multilevel analysis of the determinants and cross-national variations of HIV seropositivity in sub-Saharan Africa: Evidence from the DHS^☆^

**DOI:** 10.1016/j.healthplace.2011.06.004

**Published:** 2011-09

**Authors:** Monica Magadi, Muluye Desta

**Affiliations:** Department of Sociology, School of Social Sciences, City University London, Northampton Square, London EC1V 0HB, UK

**Keywords:** Cross-national variations, Demographic and health surveys, Determinants of HIV seropositivity, Multilevel logistic models, Simultaneous confidence intervals, Sub-Saharan Africa

## Abstract

This paper applies multilevel logistic regression models to Demographic and Health Survey data collected during 2003–2008 from 20 countries of sub-Saharan Africa to examine the determinants and cross-national variations in the risk of HIV seropositivity in the region. The models include individual-level and contextual region/country-level risk factors. Simultaneous confidence intervals of country-level residuals are used to compare the risk of being HIV seropositive across countries. The study reveals interesting general patterns in the risk of HIV seropositivity in sub-Saharan Africa. In particular, the findings highlight the gender disparity in socio-economic risk factors, partly explained by sexual behaviour factors.

## Highlights

► Interesting general patterns in the risk of HIV infection in sub-Saharan Africa. ► Gender disparity in socio-economic risk factors, partly explained by sexual behaviour. ► Background socio-economic risk factors are stronger predictors among women than men. ► Variations in the risk of HIV across countries, and to a lesser extent across regions.

## 1 Introduction

Sub-Saharan Africa remains the region most adversely affected by HIV/AIDS, accounting for 67% of HIV infections worldwide, and for 72% of world's AIDS-related deaths (UNAIDS and WHO, 2009). There are significant national variations in both scale and scope of the HIV/AIDS epidemic in Sub-Saharan Africa. According to recent UNAIDS estimates, adult national HIV prevalence is less than 2% in several countries of West and Central Africa, as well as in the horn of Africa, but in 2007 the prevalence exceeded 15% in seven southern African countries: Botswana, Lesotho, Namibia, South Africa, Swaziland, Zambia, and Zimbabwe (UNAIDS, 2008). Although there are signs that the HIV/AIDS epidemic in most of sub-Saharan Africa is stabilizing and that adult HIV prevalence appears to be falling in a growing number of countries, the actual number of people infected continues to grow (given the generally high population growth rate in the region) due to new infections and increased longevity stemming from use of anti-retroviral drugs (UNAIDS, 2010). This calls for continued efforts to improve understanding of factors associated with HIV seropositivity in the region to identify sub-population target groups for specific interventions.

A number of factors have been linked to the risk of HIV infection in sub-Saharan Africa, ranging from individual demographic characteristics (gender, age, marital status) to socio-economic status (e.g. education, wealth), cultural practices (e.g. religion, circumcision), and sexual behaviour risk factors. Available evidence suggests that women in sub-Saharan Africa are disproportionately affected in comparison with men, accounting for 60% of all HIV infections (UNAIDS, 2008). The gender disparity is particularly stark among young people. It has been noted that age shows a particularly strong association with HIV infection due to its connection to biological and psycho-social factors (Rosenthal et al., 1999; UNAIDS, 2003). In general, HIV prevalence tends to peak at a younger age for women (i.e. between the ages of 30 and 34) than for men (in late 30s and early 40s) (Gouws et al., 2008; Macro International, 2008).

Besides gender and age, another demographic factor that has been noted to show a particularly strong association with the risk of HIV infection is marital status. The Demographic and Health Survey (DHS) data from different sub-Saharan Africa countries show that individuals who are divorced, separated or widowed tend to have a considerably higher HIV prevalence than those who are single, married or cohabiting (Macro International, 2008). However, it has been noted that the relationship between marriage and the risk of HIV infection is often complex and may vary between settings or population sub-groups. For instance, while a national study of uniformed personnel in Burundi showed a higher risk (2.7 times) among married men than never-married counterparts, the prevalence of HIV was observed to be particularly high among sexually active never-married women in Lesotho, suggesting that remaining single is not universally protective against HIV infection, especially among women (UNAIDS and WHO, 2009). Among those who are married, the risk of HIV infection is likely to vary by type of marriage. For instance, the risk of infection is likely to be higher among those in polygamous unions. Available evidence suggest that concurrent partnerships dramatically increase the speed and pervasiveness of the epidemic spread (Morris and Kretzschmar, 1995), and that women with co-wives are more likely to have multiple partners (Hattori and Dodoo, 2007). Besides permitting a multiplication of sexual partners, polygamy has been linked to an accelerated transmission of sexually transmitted infections because it correlates with low rates of condom use, poor communication between spouses, and age and power imbalances (Bove and Valeggia, 2009).

Existing literature suggests mixed patterns in the association of HIV infection and education status. In the earlier years up to mid-1990s, those with the highest levels of education were found to be more likely to be infected with HIV than those at the lower end of the education spectrum (Hargreaves and Glynn, 2002). This was attributed to the fact that the more educated were more likely to be wealthier, more mobile and had broader networks of sexual partners. However, a later study by the same group of researchers revealed that the trend has been reversed in the more recent period with a lower risk of HIV infection observed among respondents with higher educational attainment (Hargreaves et al., 2008). It is possible that better awareness of the modes of HIV transmission and ways of avoiding infection during the more recent period may have led to reduced high risk behaviour among those with higher educational attainment who have higher awareness.

The association between poverty or wealth and HIV/AIDS is a complex one. Some authors have argued that the pandemic is economically opportunistic, and that poverty increases risk and vulnerability of HIV infection (Whitehead et al., 2001; Masanjala, 2007). On the other hand, it has also been argued that being wealthier may lead to reckless lifestyle and risky sexual relationship as wealthier people (particularly men) tend to attract multiple partners (Hargreaves et al., 2002; Kimuna and Djamba, 2005). Indeed, studies of the association between household/individual wealth and HIV infection based on Demographic and Health Surveys from selected countries in sub-Saharan Africa suggest that adults in wealthier households have a higher prevalence of HIV than those in poorer ones (Lachaud, 2007; Mishra et al., 2007). Rodrigo and Rajapakse (2010) noted that credible evidence exists for both arguments: while wealth shows an increased risk for both sexes, poverty places women at a special disadvantage. For women, socio-economic status may have differential effects by marital status, partner's socio-economic status, and region of residence (Wojcicki, 2005). Although there has been considerable research effort to improve understanding of the HIV–poverty/wealth link at the micro-level, the relationship between HIV prevalence and wealth/poverty at the macro-level (i.e. regional level) has received less research attention and remains unclear (Lachaud, 2007). The current study will build on previous research to establish whether the above findings generally hold across countries in SSA, paying particular attention to gender differences and macro-level socio-economic status.

Religion and circumcision are among the socio-cultural factors whose association with HIV infection have attracted considerable research attention. It has been argued that because religious leaders are esteemed and frequently exchange with the public, religion can have both positive (protective factor) or negative (against protective mechanisms such as condom use) effects on the risk of HIV infection. However, empirical evidence on the importance of religion remains weak. For instance, a study in Ghana indicated that religious affiliation had a significant effect on knowledge of HIV/AIDS, but there was no association between religious affiliation and changes in specific protective behaviour, particularly the use of condoms (Takyi, 2003). In a study of the relationship between religion and HIV risk behaviours in rural Malawi, Trinitapoli (2009) observed that although religious affiliation and involvement were not correlated with the sexual behaviour of congregation members, beliefs about appropriate sexual behaviour and particular congregational characteristics were associated with adherence to safer sex practices. Other studies have also revealed lower rates of HIV infection in some African communities where taking alcohol is prohibited as a requirement of their religious affiliation (Grey et al., 2000).

Existing biological and epidemiological evidence, including randomized trials, provide strong evidence that male circumcision significantly reduces the risk of HIV infection among men (Atashili, 2006; Weiss et al., 2009; Doyle et al., 2010, etc.). However, such findings should be interpreted with caution. It has been pointed out that even though large-scale male circumcision could avert a number of HIV infections, it is unlikely to have a major public health impact such as vaccination, and therefore should not be treated as such (Garenne, 2006). Weiss et al. (2009) noted that although there is little evidence that male circumcision directly reduces the risk of HIV in women, it does provide long-term indirect protection to women by reducing the risk of infection among heterosexual men. Unlike male circumcision, few studies have examined the link between female circumcision or female genital mutilation (FGM) and HIV infection. In a study of the relationship between male/female circumcision and prevalent HIV infection among adolescents and virgins in Kenyan, Lesotho, and Tanzanian, Brewer et al. (2007) observed that circumcised male and female virgins or adolescents were substantially more likely to be HIV infected than those who were uncircumcised. Given the recognised potential for HIV transmission through unhygienic circumcision procedures, they concluded that HIV transmission may occur through circumcision-related blood exposures in eastern and southern Africa.

**Fig. 1:**
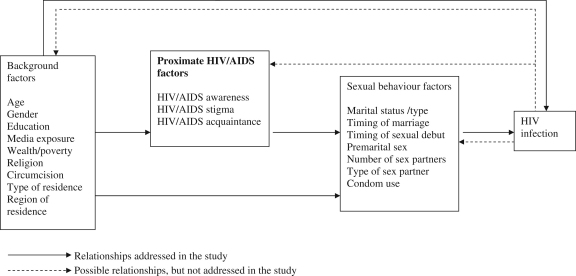
Conceptual framework for analysis of the determinants of HIV infection in sub-Saharan Africa.

*Source*: Authors' formulation.

Overall, existing studies suggest rather complex relationships between the risk of HIV infection and various background demographic, socio-economic and cultural factors such as marital status, educational attainment, wealth and circumcision. The background factors are likely to be linked to the risk of HIV infection through proximate factors relating to HIV awareness/risk perception, sexual behaviour and biological factors. Boerma and Weir (2005:s64) noted that “statistical analyses of the determinants of HIV infection that indiscriminately include underlying and proximate determinants in the same model and that do not take advantage of the multilevel data structure will produce estimates difficult to interpret”. They recommended careful examination and statistical evaluation of pathways to improve estimates of the association between determinants and transmission of HIV infection. This study places particular emphasis on the role of proximate factors such as HIV/AIDS awareness and sexual behaviour factors on the association between various background characteristics and the risk of HIV infection. The conceptual framework used to guide our analysis is presented in Fig. 1.

With heterosexual sex being the predominant mode of HIV transmission in sub-Saharan Africa (UNAIDS and WHO, 2009), sexual behaviour factors are the most proximal determinants of HIV infection in the region. Sexual behaviour is in turn influenced by a range of background socio-economic, cultural and demographic factors, either directly or indirectly through HIV/AIDS awareness. For instance, while being wealthier may directly lead to reckless lifestyle and risky sexual relationship, higher educational attainment is likely lead to greater HIV/AIDS awareness, which in turn would be expected to lead to adoption of safer sexual practices. Some of the background factors may indeed be directly linked to the risk of HIV infection due to increased vulnerability of specific sub-groups of the population, such as females. Our focus in this paper is on the direct and indirect pathways leading to HIV infection, but we recognise that HIV infection may indeed influence some of the background characteristics or proximate factors. For instance, it is possible that HIV infection may lead to: reduced wealth (e.g. due to increased medical costs or job loss); or increased HIV/AIDS awareness when individuals discover they are infected with HIV and decide to learn more about the condition; or a change in marital status when infected individuals get separated/divorced or lose partner from AIDS illness, etc. These reverse relationships will not be addressed in the study (see Fig. 1) but will be taken into account in our interpretation of the findings.

Previous research on factors associated with HIV infection in sub-Saharan Africa have largely focused on individual risk factors in specific countries. However, sociological theories have long suggested that individuals' health and behaviour is shaped not only by individual risk factors but also by the structure of the social environment in which they live. Recent developments in statistical models have made it possible to test these theories by allowing researchers to examine the additive and interactive effects of individual-level and contextual factors that affect sociological outcomes at the individual level (Moineddin et al., 2007). In particular, multilevel models have been identified as highly appropriate in assessing how context affects individual-level health risks and outcomes (O'Campo, 2003). This paper focuses on cross-national variations and overall patterns of HIV risk factors across the sub-Saharan Africa region (rather than in specific countries), as well as incorporates contextual (country-level and region-level) determinants, besides individual risk factors. While national context is important in capturing national policies and response relating to the HIV/AIDS epidemic, most of the socio-cultural and societal influences are likely to operate at sub-national (i.e. province or district) level.

### 1.1 Study objectives

We use recent Demographic and Health Survey (DHS) data collected in the mid-2000s (2003–2008) to explore individual, regional and national factors associated with HIV infection in sub-Saharan Africa. The specific objectives of this paper are to:

(i) determine individual and contextual socio-economic and demographic risk factors of HIV seropositivity among males and females in sub-Saharan Africa;
(ii) explore potential pathways of the determinants of HIV seropositivity with respect to the role of the proximate factors relating to HIV/AIDS awareness, stigma/prejudice, and sexual behaviour;
(iii) explore contextual regional (i.e. provincial) and country factors associated with HIV seropositivity; and
(iv) examine national and sub-national variations in the risk of HIV seropositivity.

The paper aims at providing an overall picture of general patterns and risk factors of HIV seropositivity in sub-Saharan Africa, useful for informing international efforts addressing the HIV/AIDS pandemic in the region. Throughout the analysis, emphasis is placed on differences between males and females, as well as cross-national variations.

## 2 Data and methods

### 2.1 The data

The paper is based on secondary analysis of existing data from the international Demographic and Health Surveys (DHS) programme from different countries in sub-Saharan Africa. The comparative nature of DHS data, along with the availability of HIV test data that can be linked to individual-level survey data, provides a unique opportunity for a population-based study of factors associated with the HIV/AIDS epidemic in different contexts. Our analysis is based on data from the DHS and AIDS Indicator Surveys (AIS) collected during the mid-2000s (between 2003 and 2008) from a total of 20 countries in Sub-Saharan Africa. A summary of the data analysed is given in Table 1.

The surveys presented in Table 1 include nationally representative samples of women and men of reproductive age (women aged 15–49 and males aged 15–54/59). Details of the sampling design and data collection procedures for each survey are available in the individual country DHS of AIS reports. The DHS or AIS HIV testing protocol undergoes a rigorous ethical review process (ICF Macro, 2010), providing for informed, anonymous, and voluntary testing of women and men of reproductive age.

### 2.2 Methods of analysis

We apply multilevel logistic regression models to explore individual and contextual regional (i.e. province) and country level factors associated with the risk of HIV seropositivity. The key outcome variable of interest is HIV seropositivity while individual-level explanatory variables include:

- background demographic, socio-economic and cultural characteristics including gender, age, urban/rural residence, educational attainment, household socio-economic status, religious affiliation and circumcision;
- HIV/AIDS factors, including awareness, stigma/prejudice, personal acquaintance with HIV/AIDS victims and previous testing for HIV; and
- sexual behaviour factors, including age at first sex, age at first union, union status/type, number of sex partners, type of sex partners and condom use.

We have included contextual country-level and regional-level factors relating to wealth index, media exposure, HIV/AIDS awareness/stigma, and sexual behaviour factors. All contextual factors are derived from relevant individual level data (with the exception of country level wealth index relating to GDP per capita^1^) based on mean indices or the proportion of the population in the region or country with specific characteristics of interest. We recognise that this limits the extent to which differences in HIV seropositivity across areas (countries or regions) would be attributable to characteristics of the areas themselves or to differences between the types of individuals living in different areas (Diez-Roux, 2001). However, the analytical approach adopted here allows for examination of area effects after controlling for relevant individual-level confounders. A description of the individual-level and contextual region/country-level variables included in the analysis is given in Table A1 in the Appendix.

Some of the explanatory variables included in the analysis (e.g. household socio-economic status, media exposure, HIV/AIDS awareness and HIV/AIDS stigma) have been derived from a set of correlated variables using principal components analysis (PCA). The PCA is a powerful tool in identifying the underlying patterns in the data and reducing the number of dimensions without much loss of information (Filmer and Pritchett, 2001). It is a useful way of creating summary indices from related sets of indicators. The resulting summary indices are linear combinations of the sets of indicator variables used to derive the PCA scores. This is the standard approach used to derive the household wealth index available in the DHS data sets (Rutstein and Johnston, 2004). In this paper, we have extended the approach to derive summary indices for media exposure, HIV/AIDS awareness and HIV/AIDS stigma/prejudice. The resulting PCA scores are then classified into various percentiles, dividing the population in each country into two, three, four or five equal parts, depending on the classification that best discriminated between the different categories with respect to HIV prevalence.

The analysis, based on pooled DHS data from 20 countries in sub-Saharan Africa, places particular emphasis on country and regional variations in factors associated with HIV seropositivity, and the extent of clustering of HIV positive individuals within countries and regions. This is necessary since an examination of the DHS data suggests considerable national and sub-national variations in HIV prevalence in sub-Saharan Africa (ICF Macro, 2010). The pooled data have a hierarchical structure with individuals nested within regions which are in turn nested within countries. In the multilevel analysis applied in this paper, countries constitute the highest (third) level (*n*=20), while regions (i.e. province) within country constitute the second level. The general form of the three-level logistic regression model used may be expressed as

(1) $$Log\;it\;\pi_{ijk}=X_{ijk}^{\prime }b+u_{jk}+v_{k}$$

where *π*_*ijk*_ is the probability of being HIV positive for an individual *i*, in the *j*th region in the *k*th country; $X_{ijk}^{\prime }$ is the vector of covariates which may be defined at the individual/household, region or country level; *β* is the associated vector of usual regression parameter estimates; and the quantities *v*_*k*_*,*and *u*_*jk*_ are the residuals at the country and region level, respectively. These are assumed to have normal distribution with mean zero and variances $\sigma_{v}^{2}\;\text{and}\;\sigma_{u}^{2}$ (Goldstein, 2003).

The estimates of country and regional level variances have been used to calculate intra-unit correlation coefficients to examine the extent to which the risk of HIV infection is clustered within countries (or regions within countries), before and after taking into account the effect of significant covariates. Since individuals within the same region are also within the same country, the intra-region correlation includes country variances (see, for example, Siddiqui et al., 1996). Thus, the intra-region (*ρ*_*u*_) and intra-country (*ρ*_*v*_) correlation coefficients are, respectively, given by

(2) $$\rho_{u}=\frac{\sigma_{u}^{2}+\sigma_{v}^{2}}{\sigma_{v}^{2}+\sigma_{u}^{2}+\sigma_{e}^{2}}$$

and

(3) $$\rho_{v}=\frac{\sigma_{v}^{2}}{\sigma_{v}^{2}+\sigma_{u}^{2}+\sigma_{e}^{2}}$$

where $\sigma_{v}^{2}$ is the total variance at country level; $\sigma_{u}^{2}$ is the total variance at province/region level; and $\sigma_{e}^{2}$ is the total variance at individual level.

For the multilevel logistic regression model, the level-1 residuals, *e*_*ijk*_, are assumed to have a standard logistic distribution with mean zero and variance ($\sigma_{e}^{2}=\pi^{2}/3$), where *π* is the constant 3.1416 (see Hedeker and Gibbsons, 1996).

The higher level residuals in multilevel analysis are useful both for diagnostic as well substantive purposes (Rasbash et al., 2005; Afshartous and Wolf, 2007). In this paper, we have used country level residuals (i.e. random effects) to explore country level variations in HIV infection by constructing 95% simultaneous confidence intervals for multiple comparisons of country effects. The country effects are presented graphically accompanied by error bars corresponding to 95% confidence intervals. Assuming the country level residuals are normally distributed with equal known standard errors, the width of the intervals to achieve a 5% significance is set at 1.39*σ* (Goldstein and Healy, 1995). Countries whose confidence intervals do not overlap are associated with different risks of HIV prevalence (significant at 5% level). The simultaneous confidence intervals are constructed before and after controlling for specific sets of individual and contextual covariates to establish which of these factors may explain the observed country risk factors.

### 2.3 Data limitations

We recognise potential data limitations that should be borne in mind while interpreting our findings. The first relates to the problem of causality since the cross-sectional nature of the data makes it impossible to determine the time sequence of key events of interest, i.e. whether the HIV infection preceded various risk factors, or whether the observed relationships are due to the effect of predisposing conditions associated with both HIV and the risk factors. Hence, we focus on the associations with HIV seropositivity, rather than causal relationships.

Secondly, we recognise possible selectivity bias due to differential non-response rates for specific sub-groups of the population. Random non-response is unlikely to create bias but selective non-response by specific high risk sub-groups may lead to bias in the observed relationships between HIV infection and respective risk factors. Coverage of HIV testing in various countries by gender and key factors presented in Tables A3(i)–(iv) in the Appendix show reasonably high response rates and no clear systematic patterns that are likely to create bias. However, it is important to exercise caution when interpreting results for specific sub-groups (e.g. urban residents or those with higher educational attainment) or countries (e.g. Malawi and Zambia) with significant refusals or overall non-response rates.

Further bias may result because HIV seropositive individuals who are in poverty are more likely to develop AIDS symptoms and die earlier, since they would be less able to afford anti-retroviral drugs. Hence, HIV-positive individuals interviewed may over-represent sub-groups of the population who are better off socio-economically. We have used the term HIV seropositivity rather than HIV infection to reflect our focus on factors associated with living with HIV infection.

Finally, an important consideration in multilevel analysis relates to sample size at the various levels. Although a consensus is yet to develop on the minimum sample size for various levels in multilevel analysis, simulation studies based on two-level linear models suggest that the number of higher level groups is more important than the number of individuals/units per group, and that the standard errors and the variance components tend to be underestimated when the number of higher level units is less than 30 (Hox, 2002; Maas and Hox, 2005). Therefore, the relatively small number of level-three units in this paper (*n*=20 countries) implies that the country-level random variances (and standard errors) may have been underestimated. More importantly, the small sample size implies low statistical power for detecting significance of country-level contextual effects.

## 3 Results

The sample characteristics and bivariate distributions of HIV prevalence by key explanatory factors are presented in Table A4 in the Appendix. We recognise that the bivariate associations may be influenced by confounding factors, associated with specific explanatory factors and the risk of infection. A multivariate analysis that simultaneously takes into account the effect of other important factors will more accurately establish the independent risk factors of HIV seropositivity.

In the multivariate analysis, we introduced the explanatory variables to the models in successive stages to establish potential pathways of the determinants, starting with background socio-economic and demographic characteristics, before introducing the proximate factors relating to HIV/AIDS awareness and finally sexual behaviour factors. The first model (Model 0) has no covariates (only the random region and country effects included); Model 1 includes only background socio-economic and demographic factors; Model 2 adds HIV/AIDS awareness and stigma factors to the background factors; while Model 3 adds the sexual behaviour factors. The results for significant factors are presented in Table 2a for females and in Table 2b for males.

With respect to individual-level background factors, the results in Table 2a suggest that across countries in sub-Saharan Africa, the highest risk of being HIV positive is observed among women who are in their early 30s, living in urban areas, have primary-level education, live in women-headed households, are not circumcised, are of non-Muslim religious affiliation, live in wealthier households or have low media exposure. The estimates across the different models suggest that the low risk of HIV seropositivity among younger females (i.e. teenagers) is to a large extent explained by sexual behaviour factors. The results suggest that the significantly higher risk of HIV seropositivity among women aged 20–24 years compared to older women of 45 years or older only becomes apparent when sexual behaviour is controlled for.

Also, the higher risk of HIV seropositivity among women in female-headed households is to a large extent explained by sexual behaviour factors. Women in female-headed households have a 72% higher odds (i.e. Exp[0.54]) of being HIV positive than their counterparts of similar background socio-economic and demographic characteristics in male headed households. However, the odds are only 15% higher (i.e. Exp[0.14]) when sexual behaviour factors relating to union status, age at first union, premarital sex, age at first sex, multiple sex partners and risky sexual behaviour (no condom use during last sex with non-spousal partner) are controlled for. This is largely attributable to the fact that some of the women in female-headed households, especially those who are themselves household heads, became widows after losing their partner to AIDS and therefore have a higher risk of being HIV positive.

There is little evidence that the other proximate factors relating to HIV/AIDS awareness/stigma or sexual behaviour factors play a significant role in the background risk factors. Contextual factors relating to media exposure (region and country level) are significant but exhibit contrasting patterns. For instance, although women in regions with relatively higher media exposure generally have a higher risk of being HIV positive, being in a country with higher media exposure is associated with a lower risk.

With respect to the proximate factors, HIV/AIDS awareness shows little association with being HIV seropositive when other factors are controlled for, but higher AIDS stigma is generally associated with a lower risk of HIV seropositivity. The results relating to sexual behaviour factors suggest that never-married women have a higher risk of HIV seropositivity than their monogamously married counterparts of similar characteristics. Women in polygamous unions also have a significantly higher risk of being HIV seropositive than their counterparts in monogamous unions. However, it is being previously married (widowed, divorced or separated) that is associated with particularly high risks of HIV seropositivity. There is no evidence that early marriage is associated with increased risk, but earlier initiation of sexual activity is associated with significantly higher risks of HIV seropositivity. As might be expected, premarital sex, multiple sex partners and risky sexual behaviour are all associated with an increased risk of being HIV seropositive.

The overall patterns of the risk of HIV seropositivity by background factors are generally similar for males (Table 2b) as for females, but one notable difference relates to living in a female-headed household which is not significant (or only marginally significant) for males despite being highly significant for females. The other notable difference relates to the patterns of HIV seropositivity by age. Even though sexual behaviour does partly explain the lower risk of HIV seropositivity among younger men compared to their older counterparts, this is to a lesser extent in comparison to women.

Some difference is also observed with respect to HIV/AIDS awareness/acquaintance factors. As in the case of women, there is no evidence of a significant association between HIV awareness and the risk of being seropositive. However, men who have personal acquaintance with an AIDS victim have a lower risk of HIV seropositivity.

The patterns of HIV risk with respect to sexual behaviour factors is generally as might be expected. Previous marriage (widowed or divorced/separated), early marriage, early initiation of sexual activity, premarital sex and multiple sex partners are all associated with a higher risk of being seropositive. However, it is interesting to note that there is no evidence of a significantly higher risk of HIV seropositivity among those engaged in ‘risky sexual behaviour' (non-condom use with non-spousal sexual partners) for men (although marginally significant for women).

### 3.1 Cross-national variations

The estimates of country and region random effects show significant variations in HIV seropositivity among both men and women across countries, and to a lesser extent across regions within countries (Tables 2a and 2b). The country variations are partly explained by background socio-economic and demographic characteristics as well as HIV/AIDS awareness/stigma factors. Estimates of intra-unit correlations suggest that about 30% of the total variation in HIV seropositivity among both males and females are attributable to country-level differences. After taking into account important background characteristics relating to educational attainment, urban rural residence, socio-economic status, media exposure, and circumcision, more than 20% (23% for females and 24% for males) of the total unexplained variation in the risk of being HIV seropositive is attributable to unobserved country level factors. This proportion reduces to about 15% when HIV/AIDS awareness and stigma factors are controlled for, but remains unchanged when sexual behaviour factors are included in the model.

**Fig. A1:**
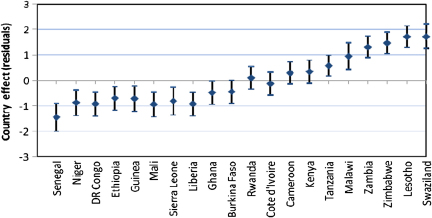
Simultaneous confidence intervals (95%) of country effects—males (Model 0).

**Fig. A2:**
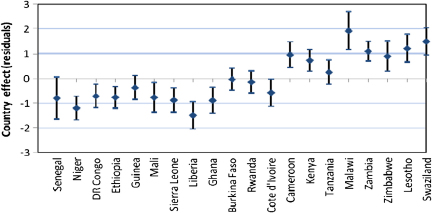
Simultaneous confidence intervals (95%) of country effects—males (Model 1).

**Fig. A3:**
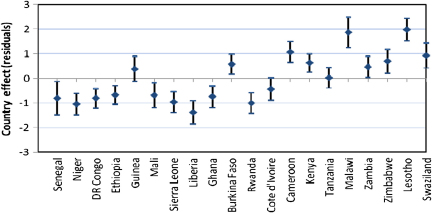
Simultaneous confidence intervals (95%) of country effects—males (Model 2).

**Fig. A4:**
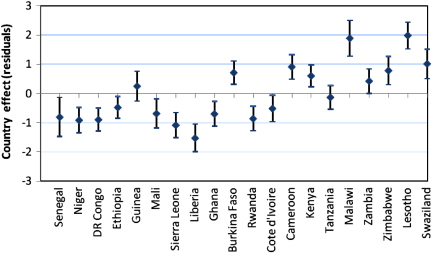
Simultaneous confidence intervals (95%) of country effects—males (Model 3).

**Fig. 2:**
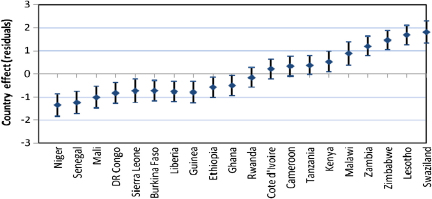
Simultaneous confidence intervals (95%) of country effects—females (Model 0).

**Fig. 3:**
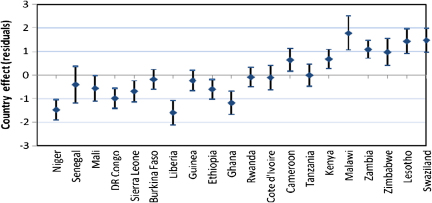
Simultaneous confidence intervals (95%) of country effects—females (Model 1).

**Fig. 4:**
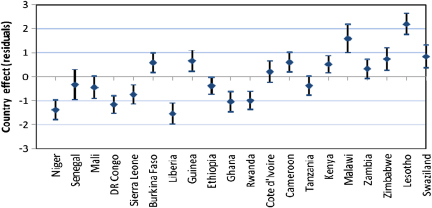
Simultaneous confidence intervals (95%) of country effects—females (Model 2).

**Fig. 5:**
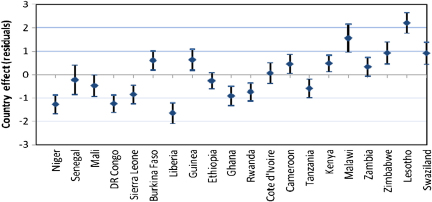
Simultaneous confidence intervals (95%) of country effects—females (Model 3).

We have used simultaneous confidence intervals (Goldstein and Healy, 1995) of country level residuals for multiple comparison of the risk of HIV seropositivity across countries, before and after controlling for different sets of factors. The countries whose 95% confidence intervals do not overlap have different risks of HIV seropositivity, significant at 5% level. As in the previous section, the first model (Model 0) has no covariates (only the random region and country effects included); Model 1 includes only background socio-economic and demographic factors; Model 2 adds HIV/AIDS awareness and stigma factors to the background factors; while Model 3 adds the sexual behaviour factors. The results for females are presented in Figs. 2–5, while corresponding figures for males are presented in Figs. A1–A4 in the Appendix. The countries are ordered from left to right by increasing HIV prevalence.

There are significant differences in the risk of HIV infection across countries in SSA. In particular, three of the Southern Africa countries (Swaziland, Lesotho and Zimbabwe) have significantly higher risks of HIV infection than all the other countries included in the analysis, except Zambia and Malawi with which the simultaneous confidence intervals overlap (Fig. 2).

Figs. 3–5 suggest that there remains a significant variation in the country risk factors after background characteristics, HIV/AIDS awareness/stigma and sexual behaviour factors are taken into account. However, the introduction of various sets of factors does modify the risk of HIV seropositivity for specific countries. In particular, controlling for background socio-economic and demographic factors (Fig. 3) leads to a notable reduction in the risk of HIV seropositivity in Liberia and Ghana, and an increase in the risk for Malawi.

Overall, women in Niger and Liberia (lowest risk) have a significantly lower risk of HIV seropositivity than their counterparts of similar socio-economic and background characteristics in all the other countries, except Senegal, DR Congo, and Ghana.

Introducing the HIV/AIDS awareness and stigma factors appears to have a notable but opposite effect on the risk of HIV infection in Rwanda and Lesotho (Fig. 4).

The risk for Rwanda is considerably reduced once the HIV/AIDS awareness/stigma factors are controlled for, such that the risk of infection is significantly lower for women in Rwanda than their counterparts of similar background and HIV/AIDS awareness and stigma characteristics in some of the countries with overall lower prevalence such as Burkina Faso and Guinea. On the contrary, the risk for Lesotho is considerably increased when the HIV/AIDS factors are controlled for, such that the risk of infection is significantly higher than all the other countries (except Malawi), including Swaziland.

Introducing the sexual behaviour factors (Fig. 5) does not considerably alter the country risk factors. As in Fig. 4, the risk of HIV infection remains highest in Lesotho and lowest in Liberia after sexual behaviour factors are controlled for.

The patterns of country risk factors observed for males (Figs. A1–A4 in the Appendix) are generally consistent with those observed for females, although the background socio-economic and demographic factors seem to have a weaker effect on the country risk factors.

## 4 Discussion, conclusions and recommendations

The main aim of this paper was to provide an overall picture of the general patterns and risk factors of HIV seropositivity across countries in sub-Saharan Africa in order to inform international efforts targeting specific population sub-groups most adversely affected by HIV/AIDS as well as identify key areas for more in-depth investigation. Overall, the results show that for both males and females, the risk of being HIV seropositive was relatively higher among urban residents, those in middle or richer households, and those who are not circumcised. These general patterns are consistent with those observed in previous studies based on DHS data from sub-samples of countries included in this paper (Mishra et al., 2007, 2009; Macro International, 2008). The analysis presented here further reveals that the risk of HIV seropositivity was significantly higher for women living in female-headed households or with primary level education compared to their counterparts in male-headed households or with no formal education. The background socio-economic factors appeared more important for HIV infection among females than males. For instance, educational attainment and gender of household head were significant for females and not males, and higher household socio-economic status was a stronger risk factor for females.

The results show mixed patterns with respect to the proximate factors relating to HIV/AIDS experience and sexual behaviour factors. There is no evidence of a significant association between HIV/AIDS awareness and HIV seropositivity, once important background socio-economic and demographic as well as other HIV/AIDS experience factors are controlled for. This suggests that the earlier finding based on bivariate analysis that ‘knowledge of all three methods of HIV prevention is associated with higher HIV prevalence' (Mishra et al., 2009:135) may be explained by differences in background characteristics. However, the finding that lower HIV/AIDS stigma at both individual and regional level are associated with a higher risk of HIV infection is consistent with patterns observed in previous studies based on bivariate analysis (Mishra et al., 2009). This suggests greater acceptance/tolerance of HIV/AIDS in settings where the epidemic is more advanced, and calls for increased efforts to address the issue of stigma/prejuduce in lower prevalence settings. The fact that men who personally know of someone living with or dead of AIDS were less likely to be HIV seropositive than their counterparts of similar background characteristics who had no personal acquintance with AIDS victims might suggest that personal acquintace with AIDS may be leading to appropriate behaviour change to avoid HIV infection.

The association between most of the sexual behaviour factors and HIV seropositivity conform to what might be expected. For both males and females, the risk was higher among the previously married (widowed, divorced or separated), those who initiated sexual activity at a younger age, had multiple sexual partners or had premarital sex. Thus, there is need for intensified efforts towards appropriate behaviour change, already observed to be effective in combating the spread of HIV transmission in selected settings in Africa (Lugalla et al., 2004).

One of the specific objectives of this paper was to identify potential pathways through which various background factors are associated with the risk of HIV infection. The results provide evidence that the proximate factors included in the analysis play a significant role in some background risk factors, especially the risk among younger women or women in female-headed households for whom sexual behaviour factors play an important role. In particular, the significanly higher risk of HIV seropositivity among women aged 20–24 years compared to older women of 45 years or older of similar background characteristics only becomes apparent when sexual behaviour factors are controlled for. Also, the strikingly high risk of HIV seropositivity among women in female-headed households, compared to their counterparts of similar characteristics in male-headed households is largely explained by sexual behaviour factors. However, this is partly attributable to the fact that some of the women in female-headed households, especially those who are themselves household heads, are widows who lost their partners to AIDS and therefore have a higher risk of being HIV seropositive.

We had postulated, from existing sociological theories, that individual's HIV risk would be affected not only by individual risk factors but by the contextual region/country factors as well. Although multilevel models have been identified as particularly useful in assessing how context affects individual-level health outcomes and in allowing examination of the additive and interactive effects of individual-level and contextual factors (Moineddin et al., 2007; O'Campo, 2003), this potential has not been fully exploited in our analysis due to limited contextual data, coupled with the relatively small number of countries in our analysis. Despite considerable random variance at country level, most of the contextual country-level factors included in the analysis were not significant, possibly due to the low statistical power for detecting significant associations, given the small number of countries included in the analysis (*n*=20).

The multilevel results show significant variations in the risk of HIV seropositivity across countries in sub-Saharan Africa, and to a lesser extent across regions within countries. About 30% of the total variation in the risk of being HIV seropositive is attributable to country-level factors. The variations across countries are partly explained by individual and contextual background socio-economic characteristics, as well as HIV/AIDS awareness/stigma factors. Controlling for background socio-economic characteristics does modify the country risk factors, especially for women. For instance, the relative risk of HIV seropositivity among women is lowered in countries such as Liberia and Ghana, but raised in Malawi, when background socio-economic factors are controlled for. This may suggest that the lower HIV prevalence observed in Malawi compared to, say, Swaziland or Lesotho, is most likely due to Malawi having higher media exposure or a higher proportion of women in the lower risk socio-economic and demographic sub-groups. On the other hand, the higher HIV prevalence observed in Liberia and Ghana, compared to countries such as Burkina Faso, may be partly attributable to the former countries having lower media exposure or a higher proportion of women in the higher risk sub-groups with respect to background characteristics. Also, the relative risk of HIV seropositivity in countries such as Lesotho and Burkina Faso are considerably increased when HIV/AIDS awareness/stigma factors are controlled for, suggesting that these countries have a disproportionately higher proportion of lower risk sub-groups with respect to HIV/AIDS awareness and stigma (e.g. high stigma).

Overall, this paper has established the general patterns in risk factors of HIV seropositivity across countries in sub-Saharan Africa, as well as identified specific areas for further investigation. The areas identified for further research include country specific as well as issue specific analyses. The patterns in country variations observed in this paper call for more in-depth country-level analysis to better understand the patterns of risk factors in individual countries, especially those that exhibit distinctive patterns when specific sets of factors are taken into account. While the general patterns for sub-Saharan Africa region are useful for informing international efforts aimed at addressing the HIV/AIDS epidemic, in-depth analyses at individual country level are particularly important for national efforts in specific countries.

The recommended issue specific research areas for further investigation include: the gender disparity in HIV infection, especially among young people; and the association between HIV seropositivity and poverty in different contexts and population sub-groups. With respect to the gender disparity, interesting differences have been noted between males and females (e.g. socio-economic factors being more important for females than males; the risk of HIV seropositivity among young females, but not males, increased when sexual behaviour factors are controlled for; never married women, but not men, have a higher risk of being HIV seropositive than married counterparts; and early marriage being associated with a reduced risk of infection for women, but an increased risk for men), all of which call for further investigation to better understand the gender disparity in HIV seropositivity and risk factors (see Magadi, 2011). Another area that has generated interesting debate and still remains to be better understood is the link between poverty and the risk of HIV infection (Holmqvist, 2009). While it has been argued that poverty increases vulnerability to HIV infection especially among women, empirical evidence presented in this paper and elsewhere (Lachaud, 2007; Mishra et al., 2007) suggest that the risk of infection is higher among individuals living in wealthier households. Further research is needed to unravel this relationship in different contexts. In particular, it would be imnportant to establish the extent to which the uban poor disadvantage that has been observed in previous studies with respect to most public health outcomes applies to the risk of HIV infection.

## Figures and Tables

**Table A1:** Description of study of variables.

**Name of variable**	**Measure**
**Outcome variable**	
HIV seropositivity	Coded as 1=if respondent tested HIV-positive; 0=otherwise. (Analysis undertaken separately for females and males)

**Individual-level variables**	
**Background factors**	
Age group (ref=45+)	Dummy variables for 5-year age groups of respondents: 15–19, 20–24, 25–29, 30–34, 35–39, 40–44, 45+.
Residence (ref=urban) rural	Coded as 1=if respondent was living in a rural area at the time of the survey; 0=otherwise
Education level (ref=none)	Dummy variables for highest educational attainment classified into three categories: none, primary, secondary.
Sex of household head (ref=male) female	Coded as 1=if respondent was living in a female-headed household at the time of the survey; 0=otherwise.
Religion (ref=catholic/orthodox)	Dummy variables for religious affiliation, re-coded into four categories: Catholic/Orthodox, Protestant/other Christian, Muslim and traditional/other.
Circumcised (ref=not circumcised)	Dummy variables for whether or not a respondent was circumcised, coded as 0=if not circumcised; 1=circumcised; 2=if information on circumcision status was not stated.
Wealth quintile (ref: lowest)	DHS household wealth index^a^ derived from information on household possessions and amenities using Principal Components Analysis (PCA). The PCA scores are classified into wealth quintiles, the lowest quintile being the poorest.
Media exposure (ref=lowest)	A composite summary index derived by PCA from information on frequency of reading newspapers, listening to radio and watching TV by respondents. The PCA scores are classified into quartiles, the lowest being equivalent to lowest media exposure.
**Proximate HIV/AIDS factors**	
HIV/AIDS awareness (ref=lowest)	A composite summary index derived by PCA from information on knowledge of modes of HIV transmission and ways to avoid being infected (See Table A2 in the Appendix). The PCA scores are classified into tertiles, the lowest being equivalent to lowest awareness.
HIV/AIDS stigma/prejudice (ref=low)	A composite index derived from respondents answers to three questions to assess presence of stigma/prejudice.^b^ The PCA scores are classified into two equal segments, with ‘low’ representing half of the population with lower HIV/AIDS stigma/prejudice.
Knows HIV/AIDS victim (ref=no)	A dichotomous variable coded as 1=if respondent personally knows someone with or dead of AIDS; 0=otherwise.
Previously tested for HIV^c^ (ref=no)	A dichotomous variable coded as 1=if respondent had been previously tested for HIV before the survey; and 0=otherwise.

**Sexual behaviour factors**	
Marital status (ref=married-monogamous)	Dummy variables for marital status/type at the time of the survey, classified into five categories: never married, married in a monogamous union, married in a polygamous union, widowed, and divorced/separated.
Age at first marriage (ref=20+)	Dummy variables for age at first marriage, classified into five categories: never married, 15 years or younger, 16–17, 18–19 and 20+.
Age at first sex (ref=20+)	Dummy variables for age at first sex, classified into five categories: never had sex, 15 years or younger, 16–17, 18–19 and 20+.
Premarital sex (ref=no)	A dichotomous variable coded as 1=if a respondent had sex before marriage, or ever had sex but never married; 0=otherwise.
Risky sexual behaviour (ref=no)	A dichotomous variable coded as 1=if a respondent had last sex with a non-spousal partner without using a condom; 0=otherwise
Multiple sex partners (ref=no)	A dichotomous variable coded as 1=if a respondent had more than one sex partner during the last 12 months before the survey; 0=otherwise

**Contextual variables**	
**Region (i.e. province) level**	
Wealth	Average household wealth index for respondent in the region
Media exposure	Average media exposure index for individuals in the region
Circumcised	Proportion of respondents in the region who are circumcised
HIV/AIDS awareness	Average awareness index for respondents in the region
HIV/AIDS stigma/prejudice	Average stigma/prejudice index for respondents in the region
Previously tested for HIV	Proportion of respondents in the region who had been previously tested for HIV before the survey.
Prevalence of polygamy	Proportion of respondents in the region in polygamous unions
Prevalence of premarital sex	Proportion of respondents in the region who had premarital sex
Multiple sex partnerships	Proportion of respondents in the region who had multiple sex partners in the 12 month preceding the survey
Risky sexual behaviour	Proportion of respondents in the region who had last sex with a non-spousal partner without using a condom

**Country level**	
Wealth	GDP per capita for country
Media exposure	Average media exposure index for individuals in the country
Circumcised	Proportion of respondents in the country who are circumcised
HIV/AIDS awareness	Average awareness index for respondents in the country
HIV/AIDS stigma/prejudice	Average stigma/prejudice index for respondents in the country
Previously tested for HIV	Proportion of respondents in the country who had been previously tested for HIV before the survey.
Prevalence of polygamy	Proportion of respondents in the country in polygamous unions
Prevalence of premarital sex	Proportion of respondents in the country who had premarital sex
Multiple sex partnerships	Proportion of respondents in the country who had multiple sex partners in the 12 month preceding the survey
Risky sexual behaviour	Proportion of respondents in the country who had last sex with a non-spousal partner without using a condom

a Rutstein and Johnston (2004).

b We have used three questions in the DHS to assess HIV/AIDS stigma/prejudice: whether the respondent would care for an AIDS patient; whether someone with HIV/AIDS should be allowed to teach; and whether they would buy vegetables from someone with AIDS. Answering no to these questions would be indicative of AIDS stigma or prejudice.

c Previous test for HIV has been included as a control variable to adjust for the fact that those who previously tested positive and knew of their HIV status may have been less likely to agree to be tested during the survey, hence, under-represented in the analysis sample.

**Table A2:** DHS questions used to derive HIV/AIDS awareness index.^a^

**Statement**	**Yes**	**No**
Ever heard of AIDS	1	0
Reduce chance of HIV/AIDS by using condoms	1	0
Reduce chance of HIV/AIDS by having only one sexual partner	1	0
HIV/AIDS can be transmitted through mosquito bites	0	1
HIV/AIDS can be transmitted by sharing utensils	0	1
A health looking person can have AIDS virus	1	0
HIV/AIDS can be transmitted through pregnancy	1	0
HIV/AIDS can be transmitted through breastfeeding	1	0

a A set of eight DHS questions (see Table A2 above) were used to construct an awareness index, through principal components analysis, and the resulting awareness score classified into tertiles. The tertiles divide the population in three equal sub-groups, where the first tertile represents the 33% of respondents with lowest awareness.

**Table A3:** (i): Coverage for HIV testing by country and gender.

	**HIV testing status**							
	**Women**				**Men**			
	**Tested**	**Refused**	**Other/missing**	**Cases**	**Tested**	**Refused**	**Other/missing**	**Cases**
Burkina Faso 2003	91.7	3.5	4.8	4575	83.9	4.5	11.6	5984
Cameroon 2004	90.4	3.7	5.9	5703	88.9	3.7	7.4	5676
Cote d'Ivoire 2005	79.1	10.6	10.3	5772	76.3	11.1	12.6	5148
DR Congo 2007	90.3	4.4	5.3	5127	86.3	5.7	8.0	4 985
Ethiopia 2005	83.2	11.2	5.6	7142	75.4	12.6	12.0	6778
Ghana 2003	89.0	4.8	6.2	5949	79.8	9.7	10.5	5345
Guinea 2005	91.8	5.7	2.5	4189	87.2	5.0	7.8	5560
Kenya 2003	76.1	12.9	11.0	4303	69.7	10.5	19.8	4183
Lesotho 2004–05	80.4	10.7	8.9	3758	67.6	13.3	19.1	3305
Liberia 2007	87.0	7.3	5.7	7448	80.4	11.3	8.3	6476
Malawi 2004	70.4	22.5	7.1	4071	63.3	21.9	14.8	3797
Mali 2006	92.0	3.2	4.8	5157	83.7	4.8	11.5	4643
Niger 2006	87.8	4.0	8.2	8738	84.2	4.7	11.1	3839
Rwanda 2005	97.0	0.9	2.1	5837	95.3	1.6	3.1	4959
Senegal 2005	84.5	9.9	5.6	5350	75.5	16.0	8.5	4375
Sierra Leone 2008	87.7	6.3	3.3	3954	85.0	7.5	4.8	3541
Swaziland 2006	87.2	9.5	3.3	5301	77.6	16.6	5.8	4675
Tanzania 2003/4	83.5	12.3	4.2	7154	77.0	13.9	9.1	6196
Zambia 2007	77.1	19.9	3.0	7408	72.2	20.1	7.7	7146
Zimbabwe 2005/06	75.9	13.2	10.9	9870	63.4	17.4	19.2	8761

**Table A3:** (ii) HIV testing response rate by age group and gender.

**Country**	**Percent of HIV sample tested by age group**							
	**Women**				**Men**			
	**15–19**	**20–29**	**30–39**	**40+**	**15–19**	**20–29**	**30–39**	**40+**
Burkina Faso 2003	90.2	91.7	92.0	93.5	85.7	83.3	86.9	87.1
Cameroon 2004	93.0	91.4	92.1	92.9	93.2	87.9	86.6	89.4
Cote d'Ivoire 2005	80.0	80.2	77.0	79.2	77.2	77.0	73.7	77.5
DR Congo 2007	89.4	91.3	91.7	92.0	86.5	86.8	86.2	89.0
Ethiopia 2005	82.3	82.8	83.9	85.0	74.1	74.4	75.3	78.1
Ghana 2003	88.9	90.1	88.3	89.5	83.3	78.6	78.9	78.7
Guinea 2005	92.5	92.1	92.1	90.8	87.8	83.1	87.6	89.9
Kenya 2003	75.3	77.1	77.8	73.9	75.9	66.2	68.6	71.1
Lesotho 2004	80.7	79.9	83.3	78.5	70.6	65.7	68.1	67.1
Liberia 2007	86.6	86.6	89.2	89.1	78.3	80.1	81.7	83.4
Malawi 2004	65.3	70.7	70.8	75.4	59.8	63.1	65.4	63.3
Mali 2006	92.0	93.3	94.4	92.7	82.4	84.0	85.7	86.3
Niger 2006	90.1	91.6	93.1	93.4	85.7	84.9	84.8	85.6
Rwanda 2005	96.2	96.8	97.8	98.8	95.6	93.6	93.8	97.1
Senegal 2005	84.8	84.7	84.3	83.8	80.9	75.2	70.9	73.5
Sierra Leone 2008	85.8	87.3	89.0	87.6	83.3	81.7	89.2	86.0
Swaziland 2006	90.4	85.4	86.4	87.5	87.7	73.7	72.5	78.0
Tanzania 2003	80.9	83.4	85.2	84.9	76.8	76.5	77.0	79.4
Zambia 2007	76.0	76.5	78.3	79.0	72.5	70.2	72.4	75.3
Zimbabwe 2005	76.5	75.5	75.9	76.3	71.4	61.2	58.3	61.8

**Table A3:** (iii) HIV testing response rate by educational attainment and gender.

**Country**	**Percent of HIV sample tested by education level**					
	**Women**			**Men**		
	**None**	**Primary**	**Sec. +**	**None**	**Primary**	**Sec. +**
Burkina Faso 2003	93.8	89.3	83.4	87.5	85.9	79.1
Cameroon 2004	95.4	93.2	89.6	90.1	91.5	88.7
Cote d'Ivoire 2005	80.8	79.3	72.7	77.0	78.6	74.1
DR Congo 2007	91.9	92.3	85.9	88.3	88.9	82.0
Ethiopia 2005	85.6	84.8	74.5	77.3	81.7	66.4
Ghana 2003	87.9	91.3	84.1	79.4	82.0	76.3
Guinea 2005	92.7	93.3	90.3	89.5	89.9	85.1
Kenya 2003	74.5	79.1	72.0	69.3	72.7	65.9
Lesotho 2004	79.4	83.9	75.9	67.0	69.7	62.2
Liberia 2007	88.7	88.7	84.6	83.3	83.4	78.3
Malawi 2004	66.9	71.8	70.0	56.7	63.6	64.5
Mali 2006	92.1	92.9	89.9	83.6	85.9	82.3
Niger 2006	92.9	89.7	71.9	85.6	85.8	72.7
Rwanda 2005	97.1	97.7	96.5	95.6	96.8	92.8
Senegal 2005	83.9	85.4	86.1	71.1	78.6	81.1
Sierra Leone 2008	88.3	86.8	86.4	87.7	87.1	81.0
Swaziland 2006	88.1	91.7	81.1	84.9	85.0	72.6
Tanzania 2003	83.3	84.7	78.2	74.9	79.4	69.1
Zambia 2007	72.9	76.7	77.6	68.1	73.1	70.6
Zimbabwe 2005	74.7	79.2	67.7	45.0	69.7	55.5

**Table A3:** (iv) HIV testing response rate by urban/rural residence and gender.

**Country**	**Percent of HIV sample tested by urban/rural residence**					
	**Women**			**Men**		
	**Urban**	**Rural**	**Cases**	**Urban**	**Rural**	**Cases**
Burkina Faso 2003	83.2	94.3	4575	69.9	89.2	5984
Cameroon 2004	88.4	95.9	5703	85.3	94.6	5676
Cote d'Ivoire 2005	74.6	83.1	5772	66.5	83.9	5148
DR Congo 2007	97.7	87.4	5127	99.7	95.3	4 985
Ethiopia 2005	72.7	88.0	7142	59.5	81.8	6778
Ghana 2003	87.6	90.5	5949	73.7	83.9	5345
Guinea 2005	88.2	94.4	4189	80.0	93.1	5560
Kenya 2003	66.2	81.7	4303	58.4	76.7	4183
Lesotho 2004–05	73.3	83.4	3758	60.7	70.2	3305
Liberia 2007	85.2	88.5	7448	75.2	84.4	6476
Malawi 2004	65.3	71.2	4071	55.7	64.8	3797
Mali 2006	89.5	93.4	5157	78.4	86.9	4643
Niger 2006	85.1	93.7	8738	77.7	89.2	3839
Rwanda 2005	95.8	97.7	5837	91.0	97.1	4959
Senegal 2005	81.9	86.6	5350	73.1	77.9	4375
Sierra Leone 2008	85.8	89.0	3954	80.0	88.9	3541
Swaziland 2006	79.7	91.2	5301	71.7	85.0	4675
Tanzania 2003/4	77.0	86.0	7154	65.0	81.6	6196
Zambia 2007	76.4	77.7	7408	67.8	75.8	7164
Zimbabwe 2005/06	65.1	82.6	9870	49.4	72.4	8761

**Table A4:** Percent of HIV positive by key characteristics among multivariate analysis sample.

**Characteristic**	**Women**		**Men**	
	**Weighted**^a^**percent HIV+**	**Unweighted cases**	**Weighted**^a^**percent HIV+**	**Unweighted Cases**
Age group	**		**	
15–19	1.5	21,099	0.9	17,130
20–24	4.4	18,553	1.6	12,793
25–29	6.7	16,155	3.8	10,889
30–34	7.6	12,914	5.8	9416
35–39	7.2	10,749	6.0	8195
40–44	6.2	8488	6.2	6542
45+	3.7	7156	3.0	11,457

Residence				
Rural	3.9	62,192	2.7	50,336
Urban	7.3	32,922	4.7	26,086

Education level				
None	2.2	36,454	1.6	19,839
Primary	6.9	33,416	3.9	27,463
Secondary+	6.9	25,244	4.0	29,120

Sex of household head				
Female	8.1	26,136	2.6	10,374
Male	4.0	68,978	3.5	66,048

Religion				
Catholic/orthodox	4.9	18,496	3.5	15,490
Protestant/other Christ.	6.7	35,079	4.0	25,470
Muslim	2.6	27,217	1.7	21,391
Traditional /other	4.8	14,322	5.2	14,071

Circumcised				
No	6.0	21,108	6.4	5577
Yes	2.5	20,851	2.2	41,907
Not stated	6.0	3155	6.2	28,938

Wealth quintile				
Lowest	2.8	17,886	2.1	13,964
Second	3.8	17,774	2.5	14,359
Third	4.4	18,461	2.9	15,067
Fourth	6.1	19,141	4.1	15,663
Highest	7.2	21,852	4.6	17,369

Media exposure				
Lowest	3.4	33,162	1.9	14,426
Second quarter	5.6	23,035	3.3	16,273
Third quarter	6.0	20861	3.6	21,281
Highest	6.5	19,056	4.1	24,442

HIV/AIDS awareness				
Low	3.6	34,523	2.9	22,161
Average	5.1	30,486	3.3	26,472
High	6.7	30,104	3.7	27,789

HIV/AIDS stigma				
Low	7.7	47,321	4.6	42,396
High	2.8	47,793	1.9	34,026

Previously tested for HIV				
No	4.6	81,578	3.0	67,554
Yes	11.1	13,536	7.2	8868

Knows someone with AIDS				
No	3.9	70,840	2.4	55,295
Yes	7.4	24,274	4.7	21,127

Marital status				
Never married	2.8	24,580	1.4	31,053
Married—mono	4.6	46,152	4.5	36,541
Married—polygamous	3.8	15,442	2.7	5517
Widowed	20.9	3817	20.3	702
Divorced/separated	11.7	5123	6.5	2609

Age at first marriage (yr)				
Never married	2.8	24,580	1.4	31,053
<16	4.1	20,901	4.6	1315
16–17	5.2	17,400	3.2	2852
18–19	6.5	13,906	4.4	5540
20+	7.8	18,327	4.8	35,662

Age at first sex (yr)				
Never had sex	0.9	14,369	0.8	14,330
<16	5.0	30,690	4.1	13,434
16–17	5.9	22,818	4.5	12,721
18–19	7.1	15,515	4.2	13,968
20+	6.1	11,722	3.5	21,969

Premarital sex				
No	3.9	72,241	1.9	46,779
Yes	8.4	22,873	5.7	29,643

Risky sexual behaviour				
No	4.7	85,133	3.5	65,277
Yes	7.9	9981	2.6	11,145

Multiple sex partners				
No	4.9	92,475	3.1	64,343
Yes	9.4	2639	4.8	12,079

**All**	**5.0**	**95,114**	**3.4**	**76,422**

*Note*: All bivariate associations presented above are significant at 1% significance level, i.e. *p*<0.01.

a Based on pooled sample weights, derived from individual country HIV sample weights and population size for respective countries.

**Table 1:** Summary of DHS in sub-Saharan Africa (SSA) analysed in the study.

**Country**	**Number of regions (i.e. provinces)**	**Women**		**Men**	
		**Cases**	**% HIV+**	**Cases**	**% HIV+**
Burkina Faso 2003	13	4189	1.8	3341	2.0
Cameroon 2004	12	5154	6.6	5041	3.9
Cote d'Ivoire 2005^a^	11	4535	6.4	3893	2.9
DR Congo 2007	11	4632	1.6	4304	0.9
Ethiopia 2005	11	5942	1.9	5107	0.9
Ghana 2003	10	5289	2.7	4265	1.6
Guinea 2005	08	3842	1.9	2925	1.1
Kenya 2003	07	3271	8.7	2917	4.6
Liberia 2007	15	6482	1.9	5190	1.2
Lesotho 2004–05	10	3020	26.4	2232	18.9
Malawi 2004	03	2864	13.3	2404	10.2
Mali 2006	09	4743	1.5	3886	1.1
Niger 2006	08	4441	0.7	3232	0.7
Rwanda 2005	12	5663	3.6	4728	2.2
Senegal 2005	11	4466	0.9	3250	0.4
Sierra Leone 2008	04	3466	1.7	3009	1.2
Swaziland 2006	04	4584	31.1	3602	19.7
Tanzania 2003–04^a^	21	5969	7.7	4774	6.3
Zambia 2007	09	5713	16.1	5161	12.3
Zimbabwe 2005–06	10	7494	21.1	5555	14.7

All (SSA)	199	95759	5.0	78,833	3.4

a AIDS Indicator Survey (AIS).

**Table 2a:** Multilevel logistic regression parameter estimates of HIV seropositivity among females in SSA (Standard errors given in brackets).

**Parameter**	**Model 0**	**Model 1**	**Model 2**	**Model 3**
***Fixed effects***				
Constant	−3.16(0.269)	−3.42(0.253)	−3.38(0.215)	−4.06 (0.222)
Age group (45 +)				
15–19		−1.23(0.072)⁎	−1.18(0.072)⁎	−0.47(0.082)⁎
20–24		0.08 (0.062)	0.07 (0.063)	0.41(0.068)⁎
25–29		0.62(0.062)⁎	0.59(0.062)⁎	0.88(0.065)⁎
30–34		0.76(0.062)⁎	0.74(0.063)⁎	0.97(0.066)⁎
35–39		0.71(0.065)⁎	0.70(0.065)⁎	0.87(0.067)⁎
40–44		0.40(0.068)⁎	0.39(0.069)⁎	0.50(0.071)⁎

Residence (urban)				
Rural		−0.51(0.040)⁎	−0.49(0.040)⁎	−0.43(0.041)⁎
Education level (none)				
Primary		0.28(0.046)⁎	0.26(0.047)⁎	0.24(0.047)⁎
Secondary +		0.14(0.054)⁎	0.08(0.054)	0.11(0.056)
Sex of household head (male)				
Female		0.55(0.029)⁎	0.54(0.029)⁎	0.14(0.033)^⁎^
Religion (Catholic/Orthodox)				
Protestant/other Christ.		−0.06(0.040)	−0.06(0.040)	−0.05(0.041)
Muslim		−0.20(0.066)^⁎^	−0.17(0.066)^⁎^	−0.14(0.067)^⁎^
Traditional/other		−0.10(0.071)	−0.06(0.072)	−0.09(0.073)
Circumcised (no)				
Yes		−0.39(0.075)⁎	−0.40(0.076)⁎	−0.37(0.076)⁎
Not stated		0.06(0.120)	0.07(0.118)	0.07(0.118)
Wealth quintile (lowest)				
Second		0.17(0.050)⁎	0.15(0.050)⁎	0.18(0.051)⁎
Third		0.26(0.050)⁎	0.24(0.051)⁎	0.27(0.051)⁎
Fourth		0.43(0.054)⁎	0.40(0.054)⁎	0.43(0.055)⁎
Highest		0.33(0.064)⁎	0.31(0.064)⁎	0.36(0.065)⁎
Media exposure (lowest)				
Second quarter		0.03(0.038)	0.01(0.039)	0.07(0.040)
Third quarter		−0.01(0.042)	−0.04(0.042)	0.03(0.043)
Highest		−0.20(0.051)⁎	−0.23(0.051)⁎	−0.13(0.052)⁎
HIV/AIDS awareness (low)				
Average			0.06(0.037)	0.04(0.037)
High			0.11(0.038)⁎	0.07(0.038)
HIV/AIDS stigma (low)				
High			−0.16(0.035)⁎	−0.16(0.035)⁎
Previously tested for HIV			0.23(0.033)⁎	0.17(0.033)⁎
Knows someone with AIDS			−0.01(0.035)	−0.02(0.036)
Marital status (married—mono)				
Never married				0.45(0.065)⁎
Married—polygamous				0.12(0.048)⁎
Widowed				1.49(0.055)⁎
Divorced/separated				0.81(0.052)⁎
Age at first marriage (20+) (yr)				
<16				−0.03(0.067)
16–17				−0.17(0.054)⁎
18–19				−0.16(0.048)⁎
Age at first sex (20+)				
Never had sex				−1.19(0.093)⁎
<16				0.26(0.061)⁎
16–17				0.30(0.054)⁎
18–19				0.22(0.050)⁎
Premarital sex				0.27(0.042)⁎
Risky sexual behaviour				0.10(0.045)⁎
Multiple sex partners				0.33(0.071)⁎

***Contextual factors—region***				
Media exposure		0.67(0.233)⁎	0.470.231)⁎	0.37(0.228)
HIV/AIDS stigma			−0.52(0.152)⁎	−0.51(0.150)⁎
Prop. tested for HIV			2.17 (0.874)⁎	2.08(0.862)⁎

***Contextual—country***				
Media exposure		−8.16(3.715)⁎	−6.89(2.982)⁎	−6.20(3.005)⁎

***Random effects***				
Region—constant	0.19(0.029)⁎	0.12(0.021)⁎	0.11(0.019)⁎	0.11(0.019)⁎
Country—constant	1.41(0.456)⁎	1.00(0.326)⁎	0.63(0.209⁎	0.64(0.211)⁎

Model 0—no covariates controlled for. Model 1—Controlling for background socio-economic and demographic factors; Model 2—Controlling for background factors plus HIV/AIDS awareness; and Model 3—Controlling for background factors, HIV/AIDS awareness, and sexual behaviour.

⁎ Statistical significance at 5% level—*p*<0.05; ns—not significant at 5% level.

**Table 2b:** Multilevel logistic regression parameter estimates of HIV seropositivity in SSA - Males.

**Parameter**	**Model 0**	**Model 1**	**Model 2**	**Model 3**
***Fixed effects***				
Constant	−3.63(0.268)	−3.10(0.273)	−3.00(0.332)	−3.37(0.234)
Age group (45+)				
15–19		−2.22(0.094)⁎	−2.18(0.094)⁎	−1.62(0.118)⁎
20–24		−1.13(0.075)⁎	−1.12(0.075)⁎	−0.86(0.086)⁎
25–29		−0.18(0.064)⁎	−0.19(0.064)⁎	−0.10(0.067)
30–34		0.38(0.061)⁎	0.37(0.061)⁎	0.39(0.063)⁎
35–39		0.54(0.063)⁎	0.53(0.063)⁎	0.54(0.064)⁎
40–44		0.55(0.067)⁎	0.54(0.067)⁎	0.54(0.068)⁎
Residence (urban)				
Rural		−0.44(0.053)⁎	−0.44(0.053)⁎	−0.42(0.053)⁎
Education level (none)				
Primary		0.12(0.064)	0.09(0.065)	0.07(0.065)
Secondary+		0.07(0.071)	0.03(0.072)	0.04(0.073)
Sex of household head (male)				
Female		0.10(0.056)	0.10(0.056)	0.12(0.057)⁎
Religion (Catholic/Orthodox)				
Protestant/other Christ.		−0.05(0.053)	−0.05(0.053)	−0.02(0.053)
Muslim		−0.13(0.091)	−0.10(0.091)	−0.14(0.067)⁎
Traditional/other		−0.10(0.069)	−0.11(0.069)	−0.08(0.091)
Circumcised (no)				
Yes		−0.39(0.095)⁎	−0.43(0.095)⁎	−0.47(0.095)⁎
Not stated		0.10(0.158)	0.06(0.155)	0.05(0.154)
Wealth quintile (lowest)				
Second		0.12(0.066)	0.12(0.066)	0.14(0.067)⁎
Third		0.22(0.067)⁎	0.21(0.067)⁎	0.24(0.067)⁎
Fourth		0.32(0.071)⁎	0.32(0.071)⁎	0.35(0.071)⁎
Highest		0.16(0.083)	0.14(0.084)	0.20(0.084)⁎
Media exposure (lowest)				
Second quarter		−0.13(0.061)⁎	−0.13(0.061)⁎	−0.11(0.062)
Third quarter		−0.03(0.061)	−0.05(0.062)	−0.04(0.062)
Highest		−0.02(0.068)	−0.04(0.069)	−0.04(0.069)
HIV/AIDS awareness (low)				
Average			0.03(0.048)	0.00(0.048)
High			0.05(0.050)	0.00(0.051)
HIV/AIDS stigma (low)				
High			−0.10(0.046)⁎	−0.10(0.046)⁎
Tested for HIV/AIDS			0.26(0.045)⁎	0.24(0.045)⁎
Knows someone with AIDS			−0.11(0.044)⁎	−0.14(0.044)⁎
Marital status (married - mono)				
Never married				−0.17(0.085)⁎
Married—polygamous				−0.10(0.086)
Widowed				1.23(0.105)⁎
Divorced/separated				0.51(0.077)⁎
Age at first marriage (20+) (yr)				
<16				0.29(0.145)⁎
16–17				0.06(0.105)
18–19				0.18(0.068)⁎
Age at first sex (20+) (yr)				
Never had sex				−0.21(0.113)⁎
<16				0.12(0.062)
16–17				0.23(0.058)⁎
18–19				0.15(0.053)⁎
Premarital sex				0.23(0.059)⁎
Risky sexual behaviour				0.08(0.060)
Multiple sex partners				0.31(0.049)⁎

***Contextual factors—region***				
Media exposure index		0.21 (0.277)	−0.06(0.276)	−0.06(0.276)
HIV/AIDS stigma			−0.57(0.182)⁎	−0.56(0.181)⁎
Prop. tested for HIV			2.21 (1.016)⁎	2.09(1.010)⁎

***Contextual—country***				
Media exposure index		−9.25(3.907)⁎	−8.18(3.040)⁎	−7.90(2.998)⁎

***Random effects***				
Region—constant	0.19(0.034)⁎	0.15(0.028)⁎	0.13(0.026)⁎	0.13(0.026)⁎
Country—constant	1.39(0.454)⁎	1.08(0.357)⁎	0.63 (0.213)⁎	0.61(0.206)⁎

Model 0—no covariates controlled for. Model 1—controlling for background socio-economic and demographic factors; Model 2—controlling for background factors plus HIV/AIDS awareness; and Model 3—controlling for background factors, HIV/AIDS awareness, and sexual behaviour.

⁎ Statistical significance at 5% level—*p*<0.05; ns—not significant at 5% level.

## Appendix A

See Tables A1–A4 and Figs. A1–A4.

## Footnotes

1 *Source*: World Bank Development Indicators database—GDP estimates for respective survey years. http://data.worldbank.org/indicator/NY.GDP.PCAP.CD?page=1 (last updated April 2011).
